# LESSONS LEARNED FROM ACCREDITING GERONTOLOGY PROGRAMS: GETTING READY!

**DOI:** 10.1093/geroni/igz038.1468

**Published:** 2019-11-08

**Authors:** Harvey L Sterns, Janet S Hahn

**Affiliations:** 1 The University of Akron, Akron, Ohio, United States; 2 Western Michigan University, Kalamazoo, Michigan, United States

## Abstract

Accreditation for Gerontology Education Council (AGEC) is an organization that collaborates with, but is independent from the Gerontological Society of America and the Academy for Gerontology in Higher Education. It is directed by a Board of Governors consisting of nine members representing higher education gerontology programs and entities associated with the field of aging. The organizational structure also includes review teams, site visitors, and staff support. Higher education degree granting programs in gerontology, specifically associate arts degree, baccalaureate degree, and master’s degree programs, are eligible to apply to AGEC for accreditation. This symposium will have presentations that focus on Overview and Experiences to Date that will describe the accreditation process and what has been learned by the accreditation of the first three degree programs. The second presentation will focus on How and Why to Apply for Accreditation and will provide background information on the steps and processes necessary to submit for the accreditation review with clarification updates. The third presentation will provide lessons learned from our first reviews with suggestions on Preparing the Self- Study and will include guidance on approaches to be taken. The fourth presentation is also lessons learned with a focus on Mapping the Competencies as part of the Self Study. Symposium presenters share important information to encourage gerontology degree programs to apply for AGEC accreditation.

